# Regulated internalization of NMDA receptors drives PKD1-mediated suppression of the activity of residual cell-surface NMDA receptors

**DOI:** 10.1186/s13041-015-0167-1

**Published:** 2015-11-19

**Authors:** Xiao-Qian Fang, Haifa Qiao, Bradley R. Groveman, Shuang Feng, Melissa Pflueger, Wen-Kuan Xin, Mohammad K. Ali, Shuang-Xiu Lin, Jindong Xu, Florian Duclot, Mohamed Kabbaj, Wei Wang, Xin-Sheng Ding, Teresa Santiago-Sim, Xing-Hong Jiang, Michael W. Salter, Xian-Min Yu

**Affiliations:** Department of Biomedical Sciences, Florida State University, Tallahassee, FL 32306 USA; Faculty of Dentistry, University of Toronto, Toronto, M5G 1G6 Canada; Department of Neurology, 1st Affiliated Hospital of Nanjing Medical University, Nanjing, 210029 PR China; BenQ Neurological Institute, Nanjing Medical University, Nanjing, 210021 PR China; Department of Neurosurgery, The University of Texas Medical School at Houston Vivian L. Smith, Houston, TX 77030 USA; Department of Physiology and Neurobiology, Medical College of Soochow University, Suzhou, 215123 China; Program in Neuroscience and Mental Health, Hospital for Sick Children, University of Toronto, Toronto, M5G 1X8 Canada

**Keywords:** NMDA receptor internalization, Ligand-gated ion channels, The function of remaining (non-internalized) surface receptors, Protein kinase D1, Phosphorylation

## Abstract

**Background:**

Constitutive and regulated internalization of cell surface proteins has been extensively investigated. The regulated internalization has been characterized as a principal mechanism for removing cell-surface receptors from the plasma membrane, and signaling to downstream targets of receptors. However, so far it is still not known whether the functional properties of remaining (non-internalized) receptor/channels may be regulated by internalization of the same class of receptor/channels. The N-methyl-D-aspartate receptor (NMDAR) is a principal subtype of glutamate-gated ion channel and plays key roles in neuronal plasticity and memory functions. NMDARs are well-known to undergo two types of regulated internalization – homologous and heterologous, which can be induced by high NMDA/glycine and DHPG, respectively. In the present work, we investigated effects of regulated NMDAR internalization on the activity of residual cell-surface NMDARs and neuronal functions.

**Results:**

In electrophysiological experiments we discovered that the regulated internalization of NMDARs not only reduced the number of cell surface NMDARs but also caused an inhibition of the activity of remaining (non-internalized) surface NMDARs. In biochemical experiments we identified that this functional inhibition of remaining surface NMDARs was mediated by increased serine phosphorylation of surface NMDARs, resulting from the activation of protein kinase D1 (PKD1). Knockdown of PKD1 did not affect NMDAR internalization but prevented the phosphorylation and inhibition of remaining surface NMDARs and NMDAR-mediated synaptic functions.

**Conclusion:**

These data demonstrate a novel concept that regulated internalization of cell surface NMDARs not only reduces the number of NMDARs on the cell surface but also causes an inhibition of the activity of remaining surface NMDARs through intracellular signaling pathway(s). Furthermore, modulating the activity of remaining surface receptors may be an effective approach for treating receptor internalization-induced changes in neuronal functions of the CNS.

**Electronic supplementary material:**

The online version of this article (doi:10.1186/s13041-015-0167-1) contains supplementary material, which is available to authorized users.

## Background

Extensive investigations have characterized mechanisms underlying constitutive and regulated internalization of cell surface proteins, which include pathways dependent or independent of clathrin or caveolae [1–3]. The internalization of cell-surface receptors or channels has been found to play important roles in regulation of immune responses, neurotransmitter release, development, and organismal homeostasis [1–4]. Internalization initiated by activating cell-surface receptors was first conceptualized as a mechanism for terminating signaling and for preventing further receptor activation by extracellular ligands. Subsequently, receptors that had undergone activity-induced internalization were found to signal [5], and to do so by engaging signaling pathways distinct from those engaged by the receptors while they were on the cell surface. However, it is not known whether the functional properties of remaining (non-internalized) receptor/channels may be regulated by internalization of the same class of receptor/channels.

The NMDAR is a principal subtype of glutamate-gated ion channel and plays key roles in neuronal plasticity and memory functions [6–10]. NMDARs are well-known to undergo two types of regulated internalization [11] – homologous and heterologous – which can be induced respectively by stimulating both the glutamate and glycine binding sites on the receptor [11, 12] and by stimulating G-protein coupled receptors such as the group 1 metabotropic glutamate receptors (mGluRs) [13–17]. In the present work, by using a combination of electrophysiological, biochemical, and genetic approaches, we determined that both types of regulated NMDAR internalization activate PKD1 signaling, which then suppresses the activity of non-internalized cell-surface NMDARs and NMDAR-mediated synaptic responses.

## Results

### Regulated internalization of NMDARs causes inhibition of single-channel activity of cell-surface NMDARs

**Fig. 1:**
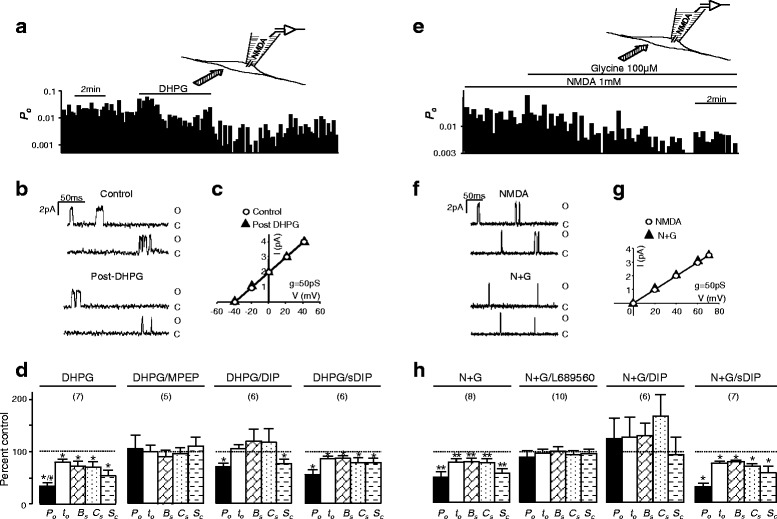
NMDAR single-channel activity is down-regulated by bath application of DHPG or high NMDA/glycine. NMDAR-mediated currents evoked by including NMDA (10 μM) and glycine (3 μM) in recording pipettes in the cell-attached configuration. Recorded NMDARs were isolated by the patch electrode from the extracellular bath environment (see cartoons in **a** and **e**). **a** an example of records of NMDA single-channel open probability (*P*_*o*_, bin: 10 s) before, during and after bath application of DHPG (50 μM). **b** examples of recorded single-channel current traces. O: Open level; C: Closed level. An example of I-V relationships before (open circles) and post (filled triangles) DHPG application is shown in (**c**). **d** summary data (mean ± SEM) showing relative changes in overall *P*_*o*_, mean open time (*t*_*o*_), and lengths of burst (*B*_*s*_), cluster (*C*_*s*_) and super-cluster (*S*_*c*_) when compared with that before the bath application of DHPG (control, dashed line). Effects of DHPG (50 μM) were also examined in neurons treated with MPEP (10 μM, DHPG/MPEP), DIP (50 μM, DHPG/DIP), or sDIP (50 μM, DHPG/sDIP). Examples of records of NMDA single-channel open probability, single-channel current traces and I-V relationships before and during bath application of a high concentration of glycine (100 μM) in the presence of 1 mM NMDA (N + G) are shown in (**e**, **f** and **g**), respectively. **h** summary data showing relative changes in channel activity when compared with that before the bath application of glycine (control, dashed line). Effects of bath application of N + G were also examined in neurons treated with L689560 (1 μM, N + G/L689560), DIP (N + G/DIP) or sDIP (N + G/sDIP). Values in brackets indicate number of patches recorded. *: P < 0.05 (paired *t*-test) in comparison with control before bath application of DHPG or glycine

Regulated NMDAR internalization causes significant reductions in whole-cell currents mediated by NMDARs [12–14] (and data not shown). Here, we questioned whether the activity of the non-internalized, residual NMDARs may be affected by NMDAR internalization. To answer this question, we recorded NMDAR single-channel activity in cell-attached patches on cultured hippocampal neurons and induced heterologous or homologous internalization of NMDARs outside the patch (see cartoons in Fig. 1a and e). Heterologous internalization was induced by treating the neurons with the group 1 mGluR agonist, (*S*)-3,5-Dihydroxyphenylglycine (DHPG, 50 μM) [13, 14]. Consistent with previous findings [13–17], application of DHPG (50 μM) caused endocytosis of both NMDA (Additional file 1: Figure S1 and Additional file 2: Figure S2) and AMPA receptors (data not shown). Homologous internalization was induced by treating with high NMDA/glycine (1 mM NMDA and 100 μM glycine, N + G) [12]. We confirmed that each treatment induced statistically significant reductions in cell-surface NMDAR proteins (see Additional file 1: Figure S1 and Additional file 2: Figure S2). Moreover, the reductions in cell-surface expression were blocked by bath application of a membrane-permeant dynamin inhibitory peptide (DIP), Myr-4–QVPSRPNRAP (50 μM, Additional file 1: Figure S1), which blocks dynamin-dependent receptor internalization [12, 18, 19]. By contrast, applying a scrambled form of DIP (sDIP, Myr-4–QPPASNPRVR, 50 μM) did not prevent the reduction in NMDAR cell-surface expression induced by DHPG or by high NMDA/glycine treatment [12, 18, 19] (Additional file 1: Figure S1).

NMDAR single-channel currents recorded in the cell-attached patch configuration were evoked with NMDA (10 μM) and glycine (3 μM) added into the patch pipette (see cartoons in Fig. 1a and e). In the cell-attached configuration, recorded NMDARs are isolated by the patch electrode from the extracellular bath environment and, therefore, will not be directly stimulated by bath-applied lipophobic agents [20, 21]. Taking advantage of this recording configuration, we investigated the activity of the isolated NMDAR single-channels before and after internalization of remote NMDARs (outside the patch) induced by bath application of DHPG (50 μM) (Fig. 1a–d) or bath application of high glycine (100 μM) in the presence of NMDA (1 mM) (Fig. 1e–h). Treatment with a high concentration of NMDA (1 mM) in the presence of a low concentration of glycine (3 μM) did not induce NMDAR internalization [12] (N in Additional file 1: Figure S1B). To prevent excitotoxic effects which can be induced by NMDA and/or glycine, a standard extracellular solution in which NaCl and KCl were replaced by Na_2_SO_4_ and Cs_2_SO_4_ was used in all of the reported biochemical and electrophysiological experiments. Consistent with previous findings [20, 22], no damage of neurons bathed with this standard solution was observed following NMDA and/or glycine application.

Before bath applying DHPG the overall channel open probability (*P*_*o*_, ratio of total open time versus recording time), mean open time (*t*_*o*_) and the duration of bursts (*B*_*s*_), clusters (*C*_*s*_) and super-clusters (*S*_*c*_) of recorded NMDAR channels (n = 11 patches) were 0.02 ± 0.007, 1.7 ± 0.2 ms, 2.8 ± 0.4 ms, 7.6 ± 1.3 ms, and 95 ± 33 ms, respectively. After the bath application of DHPG, *P*_*o*_, *t*_*o*_, *B*_*s*_, *C*_*s*_, and *S*_*c*_ were significantly reduced (Fig. 1a and d), without a change in the single-channel conductance (Fig. 1c). The group 1 mGluR antagonist, 6-Methyl-2-(phenylethynyl)pyridine (MPEP,10 μM) prevented the effects produced by DHPG (Fig. 1d). Bath application of high glycine (100 μM) in the presence of NMDA (1 mM) also caused significant reduction in NMDAR single channel activity recorded in cell attached patches (Fig. 1e and h). The glycine binding site blocker, L689560 (1 μM, Tocris, Bristol, UK), prevented the effect of glycine application (Fig. 1h).

While application of the dynamin inhibitor, DIP, alone did not significantly change NMDAR single channel activity (*P*_*o*_, *t*_*o*_, *B*_*s*_, *C*_*s*_, and *S*_*c*_ were respectively 95 ± 18 %, 96 ± 4 %, 97 ± 11 %, 112 ± 12 % and 88 ± 6 % of those prior to the DIP application), DIP co-application significantly reduced the inhibition of NMDAR channels induced by bath application of either DHPG (Fig. 1d) or glycine (Fig. 1h). sDIP had no such effect (Fig. 1d and h).

**Fig. 2:**
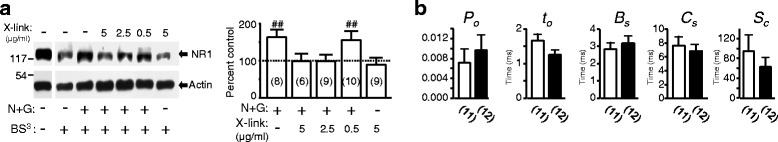
Effects of the X-link consisting of mouse GluN1 antibody and goat-anti-mouse IgG on NMDAR endocytosis and gating Blots shown in (**a**) are examples of Western analysis performed with antibodies against the GluN1 subunit (GluN1, upper blot) and actin (Actin, lower blot). The loaded cell lysates were prepared from cultured neurons treated without (“-”) or with (“+”) high NMDA/glycine (N + G: 1 mM NMDA and 100 μM glycine) in the absence (“-”) or presence (“+”) of X-link consisting of 5 μg/ml goat-anti-mouse IgG and 0.5, 2.5 or 5 μg/ml GluN1 antibody, as indicated above the blots. Immediately after the treatment, the cell membrane impermeable crosslinker, BS^3^ [Bis(sulfosuccinimidyl) suberate] , was applied to neurons at 4 °C as indicated with “+” below the blots. The ratio of the band intensity showing intracellular GluN1 subunit protein versus that of actin protein was calculated and normalized to the ratio in neurons without any treatment except BS^3^ (=100 %, control, dashed line in bar graphs). Bar graphs show summary data (mean ± SEM). ##: P < 0.01 (independent *t*-test) in comparison with control. **b** Summary data showing single-channel activity of NMDARs evoked by NMDA (10 μM) and glycien (3 μM) added into recording pipettes with (filled bars) or without (open bars) inclusion of X-link consisting of 2.5 μg/ml GluN1 antibody and 5 μg/ml goat-anti-mouse IgG

These findings implied that the activity of NMDARs in the cell-attached patch is inhibited after either heterologous or homologous internalization of remote NMDARs. However, it remained unclear whether the activity changes observed in cell-attached recordings might be induced by internalization of recorded NMDARs within the membrane patch. To address this issue, we prevented internalization of NMDARs by using an antibody complex [23, 24] (X-link), comprised of an antibody against the extracellular loop of the GluN1 subunit (mouse IgG, BD Biosciences, San Jose, CA) and a secondary antibody against mouse IgG (goat-anti-mouse IgG, G-Bioscience, St. Louis, MO). We determined that the X-link (2.5 μg/ml of GluN1 antibody and 5 μg/ml of goat-anti-mouse IgG) prevented the internalization of NMDARs induced by the bath application of high NMDA/glycine (1 mM NMDA and 100 μM glycine), but did not alter the basal level of NMDAR expression (Fig. 2a).

**Fig. 3:**
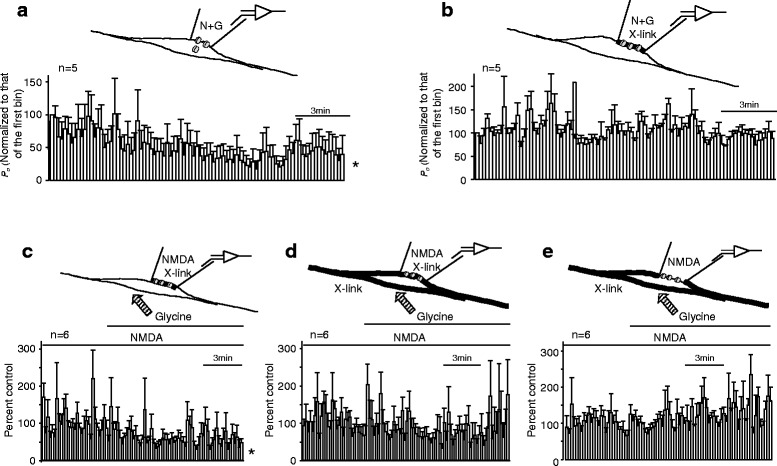
The activity of remaining surface NMDARs is down-regulated by the internalization of remote NMDARs. Averages of the normalized *P*_*o*_ (bin: 10 s) are shown in **a** - **e**. For recordings shown in (**a** and **b**), NMDAR-mediated currents were evoked by including high NMDA/glycine (N + G: 1 mM NMDA and 100 μM glycine) in recording pipettes. *P*_*o*_ recorded was normalized to the *P*_*o*_ during the first bin (control, =100 %). For recordings shown in (**c**, **d** and **e**), NMDAR-mediated currents were evoked by including NMDA (1 mM) and glycine (3 μM) in recording pipettes. *P*_*o*_ shown in (**c**, **d** and **e**) was normalized to the mean values of *P*_*o*_ (control, = 100 %) before the bath application of glycine at 100 μM. Thick lines indicate X-link applied. *: P < 0.05 (one-way ANOVA test) in comparison with *P*_*o*_ of the first bin in (**a**) or with control before bath application of glycine in (**c**)

We then examined the activity of surface NMDARs when these channels were immobilized by the X-link. Inclusion of the X-link in the recording pipettes did not significantly affect the activity of recorded NMDAR channels (Fig. 2b). Inclusion of high NMDA/glycine (N + G) in the recording pipette caused a decline in NMDAR channel *P*_*o*_ recorded in cell-attached patches (Fig. 3a), implying the internalization of recorded NMDARs. Such reductions were not observed when the X-link was included in recording pipette containing high NMDA/glycine (1 mM NMDA and 100 μM glycine) (Fig. 3b), suggesting that the X-link added into the recording pipettes immobilized surface NMDARs recorded and thereby prevented the *P*_*o*_ reduction.

We then recorded NMDAR single channel currents evoked by including a high concentration of NMDA (1 mM) and a low concentration of glycine (3 μM), which does not cause NMDAR internalization [12] (N in Additional file 1: Figure S1B), in the pipette solution containing the X-link (Fig. 3c). We found that bath application of glycine at the high concentration (100 μM) capable of inducing NMDAR internalization still resulted in a reduction in *P*_*o*_ of recorded channels which had been immobilized with the X-link (Fig. 3c). In contrast, bath application of the X-link, which immobilized remote NMDARs, prevented *P*_*o*_ reduction in neurons recorded with pipettes filled either with (Fig. 3d) or without (Fig. 3e) the X-link. Thus, the inhibition of NMDARs recorded in cell-attached patches following bath application of glycine at 100 μM was unlikely to be induced by the internalization of recorded receptors within membrane patches but instead by the internalization of remote NMDARs. Taken together, our data demonstrate that regulated internalization of cell surface NMDARs not only reduces the number of NMDARs expressed on the cell surface but also causes an inhibition of the activity of non-internalized NMDARs.

### Regulated NMDAR internalization causes increased serine phosphorylation of surface NMDARs

**Fig. 4:**
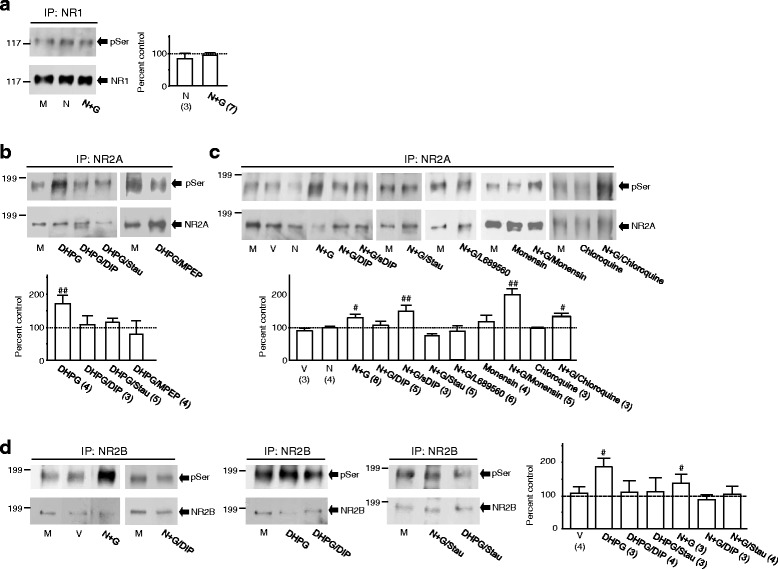
NMDAR internalization causes increases in serine phosphorylation of surface NMDARs. Blots shown in (a - d) are examples of experiments, in which NMDA GluN1 (**a**), GluN2A (**b** and **c**) or GluN2B (**d**) proteins was immunoprecipitated from the synaptic plasma membrane (LP1) of cultured neurons treated with agents in bath as indicated underneath the blots. The same filters were then stripped off and successively probed with anti-phosphoserine antibody (pSer, upper blots) and NMDAR antibodies (lower blots) for the GluN1 (**a**), GluN2A (**b** and **c**) or GluN2B (**d**) subunit. The ratio of band intensity showing phosphorylated versus that of total GluN1, GluN2A or GluN2B subunit proteins was normalized to the ratio in neurons treated only with culture medium (control, = 100 %, dashed line in bar graphs). Bar graphs show summary data (mean ± SEM). M: culture medium (M), V: vehicle, N: NMDA (1 mM), N + G: high NMDA/glycine (1 mM NMDA and 100 μM glycine). Effects of DHPG (50 μM) were also examined in neurons treated with DIP (50 μM, DHPG/DIP), staurosporine (1 μM, DHPG/Stau) or MPEP (10 μM, DHPG/MPEP); Effects of N + G were also examined in neurons treated with DIP (N + G/DIP), sDIP (N + G/sDIP), staurosporine (1 μM, N + G/Stau), L689560 (10 μM, N + G/L689560), monensin (10 μM, N + G/momemsin) or chloroquine (200 μM, N + G/chloroquine). #, ##: P < 0.05, 0.01 (Independent *t*-test) in comparison with control (M). Values in brackets indicate the number of experimental repeats

Phosphorylation regulation has been recognized as an important mechanism underlying the regulation of NMDA receptor trafficking and channel functions [25–29]. To understand mechanisms underlying the activity regulation of non-internalized cell surface NMDARs by NMDAR internalization, we examined remaining NMDARs located at the synaptic plasma membrane (LP1) [24, 30, 31] of neurons after NMDAR internalization. When compared with that in neurons treated only with culture medium (control), no significant change in serine phosphorylation was detected in the GluN1 subunit following the treatment with high NMDA/glycine (Fig. 4a). However, the serine phosphorylation of the GluN2A subunit was significantly increased following either DHPG (Fig. 4b) or high NMDA/glycine application (Fig. 4c). No such increase was induced by DHPG in neurons treated with DIP (Fig. 4b). Compared with controls, no phosphorylation increase was induced by vehicle or NMDA (1 mM) in the presence of a low concentration of glycine (3 μM) or by high NMDA/glycine in neurons treated with DIP (50 μM), staurosporine (1 μM) or L689560 (10 μM) (Fig. 4c). The serine phosphorylation increase was not affected by sDIP (50 μM). Either monensin (10 μM) or chloroquine (200 μM), blocking receptor recycling pathways [32–34], produced no effect on the phosphorylation increase induced by bath application of high NMDA/glycine (Fig. 4c). Application of monensin or chloroquine alone did not induce any significant changes in the serine phosphorylation either (Fig. 4c). Serine phosphorylation in the GluN2B subunit was also increased following application of either high NMDA/glycine or DHPG. These increases were prevented by either DIP or staurosporine (Fig. 4d).

To examine the mechanisms underlying internalization-induced serine phosphorylation, recombinant GluN1-1a/GluN2A or GluN1-1a/GluN2B receptors were expressed in HEK293 cells and used to identify the site(s) of NMDAR phosphorylation. We found that after 10 min treatment with high NMDA/glycine there were significant reductions in surface expression of these receptors (Additional file 3: Figure S3A and B). A significant reduction was also noted in surface GluN1-1a/GluN2A1-857 receptors (Additional file 3: Figure S3C), in which the C-tail of the GluN2A subunit was truncated immediately after aa 857. However, no significant reduction in surface GluN1-1a/GluN2B1-857 receptors was observed, in which the C-tail of the GluN2B subunit was truncated after aa 857 (Additional file 3: Figure S3D). These findings indicate that internalization of GluN2B-containing receptors is prevented by the C-tail truncation.

Similar to NMDARs in cultured neurons (see Fig. 4b–d), we found significant increases in serine phosphorylation in both wild-type GluN2A and GluN2B subunits after NMDAR internalization in receptors expressed heterologously (Additional file 4: Figure S4A and B). However, such increases were not detected in either GluN2A1-857 or GluN2B1-857 mutants (Additional file 4: Figure S4A and B). These findings show not only that internalization of NMDARs is tightly regulated by subunit-specific mechanisms [35–38], but also provided another line of evidence suggesting that the event of NMDAR internalization acts as an upstream signal activating downstream pathway(s) that, in turn, phosphorylate(s) remaining surface NMDARs.

We then generated truncation mutations in the GluN2A C-tail immediately after aa 1396, 1415, 1419, or 1424, respectively (Additional file 4: Figure S4C), and found that serine 1416 and 1420 residues might be the targets of phosphorylation in the GluN2A subunit (Additional file 4: Figure S4D). By mutating these serines to phenylalanines, we identified that serine 1416 (S1416) in the GluN2A subunit is a key residue being phosphorylated following internalization of GluN1-1a/GluN2A receptors in HEK293 cells (Additional file 4: Figure S4E). To confirm this mechanism in neurons, an antibody selectively recognizing phosphorylated S1416 (pS1416) in the GluN2A subunit (anti-pS1416) was raised (Additional file 5: Figure S5A–D). A significant increase in serine phosphorylation of the GluN2A subunit was detected with anti-pS1416 after NMDAR internalization in cultured neurons (Additional file 5: Figure S5E). This established that the event of NMDAR internalization causes increases in serine phosphorylation of remaining surface NMDARs in neurons.

### The increase in NMDAR phosphorylation is induced by PKD1 activation

**Fig. 5:**
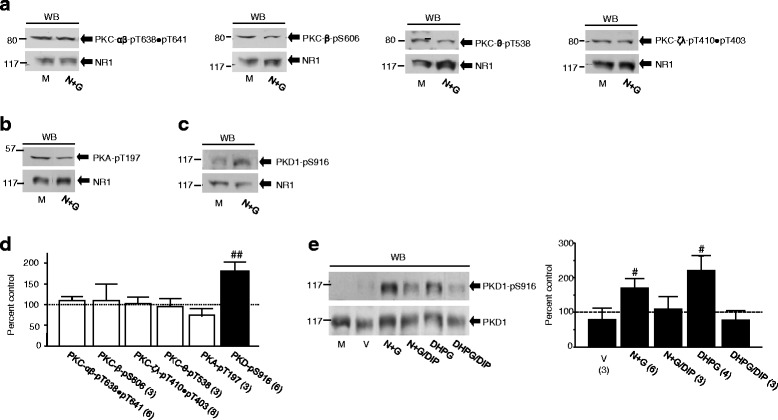
The event of NMDAR internalization activates PKD1. Upper blots (from left to right) shown in (**a**) represent examples of Western analysis with an antibody for phosphorylated T638 and T641 in PKC-α and β, phosphorylated S606 in PKC-β, phosphorylated T538 in PKC-θ or phosphorylated T410 and T403 in PKC-ζ and λ. Upper blots in (**b** and **c**) show examples of experiments with antibodies recognizing phosphorylated T197 in PKA and phosphorylated S916 in PKD1, respectively. Lower blots in (**a** - **c**) show GluN1 proteins detected in each experiment. The ratio of band intensity showing phosphorylated enzymes versus that showing GluN1 subunit protein detected in the same lysates was normalized with the ratio in neurons treated with culture medium (M, control, =100 %, dashed line in **d**). Bar graphs in (**d**) show summary data (mean ± SEM) of the normalized ratios. For blotting analysis shown in (**e**), PKD1 was immunoprcipitated with an antibody recognizing both phosphorylated/active and non-phosphorylated/inactive PKD1 from neurons treated with agents in bath as indicated. The same filters were stripped and successively probed with antibodies recognizing active PKD1 (upper blot) and total PKD1 (lower blot). The ratio of band intensity of active versus that of total PKD1 was normalized to the ratio in neurons treated only with culture medium (M, control). Bar graphs show summary data. #, ##: P < 0.05, 0.01 (independent *t*-test) in comparison with control

A large number of studies have shown that PKC and PKA may phosphorylate NMDARs at serine residues and thereby modulate NMDAR activity [25, 27, 39–48]. We thus examined whether PKA or any one of the PKC isoforms including PKCμ (which is now classified as PKD1 [49, 50]) might act as a downstream factor activated by the event of NMDAR internalization. The activation status of PKC isoforms [51–54] (Fig. 5a and d), PKA [55] (Fig. 5b and d) or PKD1 [56] (Fig. 5c and e) was thus examined with specific antibodies which selectively detect the enzyme activity-related autophosphorylation. No significant change in the phosphorylation of these enzymes was found after NMDAR internalization except for PKD1 (Fig. 5). Blocking NMDAR internalization prevented the increase in phosphorylated PKD1 (Fig. 5e).

To determine the role of PKD1 in NMDAR phosphorylation, we examined the effects of PKD1 immuno-purified from neurons *in vitro* (Additional file 6: Figure S6A and B). The GluN2A C-terminal fragment protein (aa 1096–1464, 5 μg), which was purified from a bacterial expression system [57, 58], was incubated in vitro with a similar amount of PKD1 immuno-purified respectively from neurons treated with culture medium, vehicle or high NMDA/glycine (Additional file 6: Figure S6A). Incubation with PKD1 purified from neurons treated with high NMDA/glycine caused significantly elevated serine phosphorylation of the GluN2A C-terminal fragment when compared with those induced by PKD1 purified from neurons treated with culture medium or vehicle (Additional file 6: Figure S6B).

Furthermore, we found that the application of recombinant PKD1 protein in active form (SignalChem, Richmond, BC) into neurons produced a dose-dependent inhibition of NMDAR currents recorded in the whole-cell configuration (Additional file 6: Figure S6C). Similar effects of PKD1 were also found on GluN1-1a/GluN2A or GluN1-1a/GluN2B receptors expressed in HEK cells (Additional file 6: Figure S6D and E). This inhibitory effect of PKD1 was prevented by S1416F mutation in GluN2A (Additional file 6: Figure S6D) or by C-tail truncation from aa 858 in the GluN2B subunit (Additional file 6: Figure S6E). Thus, we conclude that NMDARs can be phosphorylated and thereby down-regulated by PKD1. We then hypothesized that knockdown of PKD1 in neurons would reduce both the NMDAR phosphorylation and the inhibition of NMDAR activity induced by NMDAR internalization if endogenous PKD1 indeed acted as a critical downstream factor activated by the event of NMDAR internalization.

### Knockdown of PKD1 does not affect NMDAR internalization, but prevents both the enhancement of NMDAR phosphorylation and the inhibition of remaining (non-internalized) surface NMDARs induced by NMDAR internalization

**Fig. 6:**
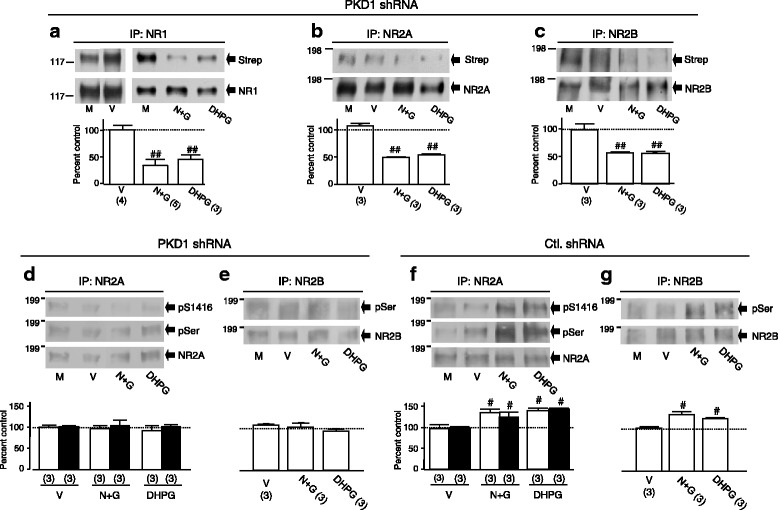
Knockdown of PKD1 does not affect NMDAR internalization but prevents the NMDAR internalization-induced increases in serine phosphorylation of surface NMDARs. **a** - **c** DHPG or high NMDA/glycine (N + G) induced NMDAR internalization in neurons infected with PKD1 shRNA. After biotinylation of these neurons, GluN1 (**a**), GluN2A (**b**) or GluN2B (**c**) subunit protein was immunoprecipitated. The same filters were stripped and successively probed with HRP-conjugated streptavidin (Strep, upper blot) and an antibody against the GluN1 (**a**), GluN2A (**b**) or GluN2B (**c**) subunit (lower blots). Bar graphs show summary data (mean ± SEM) of the normalized ratios between biotinylated and total GluN1, GluN2A or GluN2B subunit proteins detected. NMDAR phosphorylation induced by bath application of DHPG or N + G in neurons infected with PKD1shRNA is shown in (**d** and **e**). NMDAR phosphorylation in neurons infected with control shRNA (Ctl. shRNA) is shown in (**f** and **g**). The same filter shown in (**d** or **e**) was stripped and successively probed with anti-pS1416 (top blots), anti-pSer (middle blots) and GluN2A antibody (bottom blots). The anti-pSer (upper blots) and GluN2B antibody (lower blots) were respectively used to probe the filters shown in (**e** and **g**). Bar graphs show summary data (mean ± SEM) of the normalized ratios between phosphorylatd and total GluN2A (**d** and **f**) or GluN2B (**e** and **g**) protein. Open and filled bars in (**d** - **g**) show changes detected with anti-pSer and anti-pS1416 antibodies, respectively. #, ##: P < 0.05, 0.01 (independent *t*-test) in comparison with control

We found that infection with PKD1 shRNA (Open Biosystems/Thermo Fisher Scientific, Lafayette, CO; Additional file 7: Figure S7A) selectively reduced the expression of endogenous PKD1 without affecting the expression of other proteins such as PKC-α (Additional file 7: Figure S7B) when compared with those following infection with control shRNA (Additional file 7: Figure S7B). Compared with those found in neurons without infection with shRNA (see Additional file 1: Figure S1), the knockdown of PKD1 did not produce any change in the internalization of NMDARs induced by treatment with DHPG or with high NMDA/glycine (Fig. 6a–c). However, PKD1 shRNA infection prevented the increases in the serine phosphorylation of both the GluN2A (Fig. 6d) and GluN2B (Fig. 6e) subunits whereas infection of control shRNA did not alter the increases in the serine phosphorylation (Fig. 6f and g) induced by DHPG or high NMDA/glycine treatment.

**Fig. 7:**
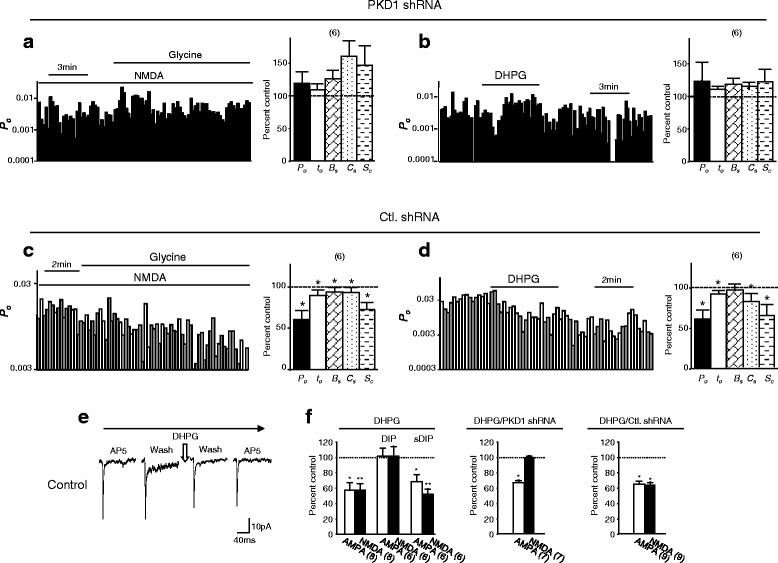
Knockdown of PKD1 blocks the NMDAR internalization-induced inhibition of remaining surface NMDARs. **a** and **b** show examples of records of *P*_*o*_ (bin: 10 s) and summary data (Bar graph) of relative changes in overall *P*_*o*_, *t*_*o*_, *B*_*s*_, *C*_*s*_ and *S*_*c*_ when compared with that before bath application of 100 μM glycine (**a**) or 50 μM DHPG (**b**) (control, dashed line) in neurons infected with PKD1 shRNA. **c** and **d** show examples of records of *P*_*o*_ and summary data of relative changes in NMDAR channels activity in neurons infected with control shRNA (Ctl. shRNA). **e** shows examples of averaged mEPSC traces recorded from neurons before and after bath application of DHPG. Bar graphs in **f** show relative changes in AMPAR and NMDAR components of mEPSCs when compared with control (dashed line) before bath application of DHPG.  *,**P < 0.05, < 0.01 (paired *t*-test) in comparison with control before glycine or DHPG application

We then examined the effects of knocking down PKD1 on NMDAR-mediated single channel activity (Fig. 7). In neurons infected with PKD1 shRNA, *P*_*o*_, *t*_*o*_, *B*_*s*_, *C*_*s*_, and *S*_*c*_ of recorded NMDARs (n = 6 patches) were 0.033 ± 0.013, 1.5 ± 0.07 ms, 2.3 ± 0.2 ms, 5.9 ± 1.4 ms, 53.1 ± 21 ms, respectively. Consistent with findings in neurons treated with staurosporine (1 μM) (data not shown), no significant reduction in *P*_*o*_, *t*_*o*_, *B*_*s*_, *C*_*s*_ or *S*_*c*_ was found after bath application of 100 μM glycine in the presence of 1 mM NMDA (Fig. 7a) or DHPG at 50 μM (Fig. 7b). In contrast, the bath application of glycine or DHPG produced significant inhibition of NMDAR activity recorded in neurons infected with control shRNA (Fig. 7c and d). These data evidenced that the knockdown of PKD1 prevented both the increase in serine phosphorylation and the inhibition of remaining surface NMDARs following internalization of remote NMDARs.

**Fig. 8:**
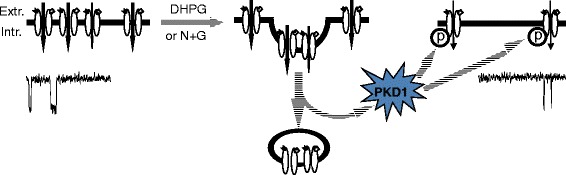
Effects of regulated NMDAR internalization. A diagram shows that the regulated internalization of cell surface NMDARs not only reduces the number of NMDARs expressed on the cell surface but also causes phosphorylation and an inhibition of remaining (non-internalized) surface NMDARs through PKD1. Extr.: Extracellular, Intr.: Intracellular

To determine the functional significance of the modulation of non-internalized surface NMDARs, miniature excitatory post-synaptic currents (mEPSCs) in cultured hippocampal neurons without or with knockdown of PKD1 were recorded (Fig. 7e and f; Additional file 8: Figure S8). Consistent with previous studies [13–16], bath applying DHPG significantly reduced both the NMDAR and AMPAR-mediated mEPSC components in neurons without shRNA infection (Control, Fig. 7e and f; Additional file 8: Figure S8). DIP, which was applied into neurons through inclusion of the peptide in the intracellular solution filling recording pipettes, prevented the inhibition of both the NMDAR-and AMPAR-mediated mEPSCs following DHPG application (Fig. 7e and f). Interestingly, PKD1 knockdown blocked the inhibition of mEPSCs induced by NMDAR internalization but did not affect the effect induced by AMPAR internalization (Fig. 7e and f; Additional file 8: Figure S8D–F). Infection with control shRNA did not produce any effect (Fig. 7e and f; Additional file 8: Figure S8G - I). These findings not only strongly suggest that PKD1 may be selectively activated by NMDAR internalization but also demonstrate that the activity of remaining surface NMDARs plays an important role in the regulation of NMDAR-mediated synaptic activity by NMDAR internalization. Altogether, we conclude that regulated NMDAR internalization causes down-regulations of the activity of remaining (non-internalized) surface NMDARs and the synaptic transmission through serine phosphorylation of remaining surface NMDARs induced by PKD1 activation (Fig. 8).

## Discussion

In order to determine whether the functional properties of remaining surface NMDARs, which are not internalized, may be altered by internalization of remote NMDARs, we conducted single channel recordings in the cell-attached configuration. Previous studies have demonstrated that NMDAR activity can be regulated by remote NMDARs through intracellular signaling pathways [20, 21, 59]. The current work demonstrates that internalization of remote NMDARs causes changes in the activity of NMDARs remaining on the cell surface. This conclusion is also supported by the findings that the application of the protein kinase inhibitor staurosporine (data not shown), or the knock-down of PKD1 did not prevent NMDAR internalization but did block the increase of serine phosphorylation in NMDARs and the inhibition of their activity. If the inhibition of NMDAR channels recorded within membrane patches following the bath application of DHPG or high NMDA/glycine were caused by internalization of the recorded NMDAR channels, the bath application of either DHPG or high NMDA/glycine to neurons treated with either staurosporine or PKD1 shRNAs would still induce down-regulations of NMDA channel activity recorded within membrane patches.

It was thought until now that through reducing the number of active receptor/channels located on the cell surface, the regulated receptor/channel internalization reduced the receptor/channel-mediated responses. Our single channel data showed that the NMDAR internalization, which reduced the amount of NMDARs expressed on the neuronal surface, also led to inhibition of the activity of remaining surface NMDARs. Furthermore, the inhibition of activity was mediated through intracellular PKD1 signaling as we found that knockdown of PKD1 prevented the inhibition of single channel activity of remaining NMDARs induced by NMDAR internalization, and also prevented the reduction of synaptic NMDAR responses (Fig. 7f). Thus, our findings imply a novel mechanism to reduce NMDAR-mediated responses following NMDAR internalization in addition to decreased cell-surface expression of the receptors. The inhibition of remaining surface NMDARs also plays a critical role in the regulation of NMDAR-mediated responses by NMDAR internalization.

A large amount of data have demonstrated that the activation of group 1 metabotropic glutamate receptors through application of DHPG is an important mechanism underlying the induction of long-term depression of AMPAR-mediated synaptic responses (LTD) [10, 14–17, 60]. More detailed studies have shown that phosphorylation of the GluA1 subunit of AMPARs by PKA may promote AMPAR surface insertion and decreases the endocytosis of the receptor, leading to increases in AMPAR on cell surface [61]. Two receptor pathways are proposed to drive the endocytosis of distinct populations of AMPARs: 1) NMDARs activation induces the endocytosis of rapidly cycling surface AMPARs not directly associated with GRIP1/2; 2) mGluR activation induces the endocytosis of non-cycling GRIP-bound surface AMPARs [60]. DHPG-induced LTD may, or may not, require activation of NMDA receptors, depending upon the experimental conditions [10, 17].

NMDARs are well-known to undergo two types of regulated internalization [11] – homologous and heterologous. Homologous internalization can be induced by treating with high NMDA/glycine, which induces internalization of NMDARs but not other receptors such as AMPARs [12]. Heterologous internalization can be induced by application of DHPG, which may also induce endocytosis of AMPARs and mGluRs [13–17]. Our data showed that through the activation of PKD1, remaining surface NMDARs were inhibited by regulated endocytosis of NMDARs in heterologous and also in homologous models. Moreover, in the heterologous model the knockdown of PKD1, had no effect on the inhibition of AMPAR-mediated responses but significantly blocked the inhibition of NMDAR-mediated responses. Thus, PKD1 signaling activated by regulated NMDAR endocytosis appears to differentially inhibit NMDARs but not on AMPARs.

The phenomenon discovered in our work underlying the regulation of remaining surface receptor/channels by the endocytosis of the same type receptor/channels might therefore be NMDAR-specific. Detailed mechanisms underlying how PKD1 signaling is activated by NMDAR endocytosis and what role, if any, is played by other receptors such as AMPAR in the PKD1 activation remains to be clarified. Further clarifying the mechanisms underlying the regulation of remaining surface receptor/channels is challenging and required for understanding of the principal biological event: internalization of cell-surface receptor/channels. Altogether, we conclude that the regulated NMDAR internalization causes down-regulations of the activity of remaining (non-internalized) surface NMDARs and the synaptic transmission through serine phosphorylation of remaining surface NMDARs induced by PKD1 activation (Fig. 8).

Previous studies, including ours, have shown that tyrosine phosphorylation may enhance NMDAR activity while tyrosine dephosphorylation may down-regulate NMDAR activity [62–66] and trigger NMDAR internalization [37, 67–71]. Striatal-enriched protein tyrosine phosphatase (STEP) has been identified as an important tyrosine phosphatase promoting NMDAR endocytosis [37, 67–71]. The tyrosine residue 1472 in the GluN2B subunit has been clarified as a critical site underlying the regulation of NMDAR trafficking by tyrosine phosphorylation [65, 66, 72]. In order to identify mechanisms underlying the NMDAR endocytosis-induced gating inhibition of remaining cell surface NMDARs, we required a model in which the regulated receptor endocytosis will not be altered. Since changes in tyrosine phosphorylation may alter NMDAR endocytosis [37, 65–72], and a significant enhancement in serine phosphorylation of NMDARs on the cell surface was noted following regulated NMDAR endocytosis, in this work we focused on the effects produced by phosphorylation at serine residue(s) of NMDARs. Thus, roles played by tyrosine phosphorylation in the regulation of remaining surface NMDARs remains to be clarified.

C-termini of NMDAR subunits are known to be critically involved in regulation of both the NMDAR internalization and gating [36–38, 73, 74]. The present work provided direct evidence showing that the C-tail portion after aa 857 in the GluN2B, but not in the GluN2A subunit, is required for the regulated NMDAR internalization. Taking advantage that the truncation of the C-tail after aa 857 did not affect the internalization of GluN1-1a/GluN2A1-857 receptors, S1416 in the GluN2A subunit was identified to be critically involved in the serine phosphorylation and gating regulation induced by NMDAR internalization. In contrast, the portion after aa 857 in the GluN2B subunit is likely involved in both the regulated NMDAR internalization and the internalization-induced gating regulation. Since the truncation after aa 857 in the GluN2B subunit blocked the internalization of GluN1-1a/GluN2B1-857 receptors, the residue(s) involved in the increased serine phosphorylation in the GluN2B subunit induced by NMDAR internalization still needs to be characterized.

Taking together with findings that the application of active recombinant PKD1 protein into cells produced inhibition of NMDAR currents and that this inhibition was blocked by S1416F mutation in the GluN2A subunit or C-tail truncation in the GluN2B subunit, we conclude that regulated NMDAR internalization acts as an upstream signal which activates the downstream PKD1 and thereby causes the phosphorylation and down-regulation of remaining surface NMDARs (Fig. 8). PKD1 is a ubiquitously expressed serine/threonine kinase, and was originally identified as PKC-μ [49, 50], an atypical isoform of PKC. PKD1 is found to play important roles in fission of vesicles at the Golgi compartment, coordination of cell migration and invasion, regulation of gene transcription and surface protein translocation [49, 50, 75, 76]. PKD1 can be activated through multiple mechanisms including PKC phosphorylation [77], activation of G-protein-coupled receptors [49, 78, 79] and ligand-gated channels [80]. The C-terminal autophosphorylation sites in PKD1 is part of the PDZ binding motif of postsynaptic density-95 (PSD95), which thereby provides a mechanism for PKD1 to interact with proteins located at neuronal surface [81]. In neurons PKD1 has been found to be involved in the regulation of dendritic trafficking and the establishment of neuronal polarity [49, 50], and the PKD homolog Dkf-2 is found to be involved in the regulation of associative learning and memory in *Caenorhabditis elegans* [82].

The NMDAR internalization-induced inhibitions of both the remaining surface NMDAR activity and the NMDAR-mediated synaptic response can be significantly reduced by knockdown of PKD1. This finding has not only revealed a role played by remaining surface NMDARs in the regulation of NMDAR functions by NMDAR internalization, but also provided a novel mechanism that quantity changes in cell surface receptors may down-regulated the receptor-mediated whole-cell response through depressing the activity of non-internalized surface receptors.

## Conclusion

Thus, we conclude that regulated internalization of cell surface NMDARs not only reduces the number of NMDARs expressed on the cell surface but also causes an inhibition of the activity of non-internalized NMDARs through the activation of PKD1 (Fig. 8). The function of remaining surface NMDARs play critical roles in the regulation of neuronal activity by NMDAR endocytosis. As such, further clarifying the signaling pathway(s) underlying NMDAR internalization-induced PKD1 activation and the mechanism and function of the regulation of remaining surface receptors will be essential for developing new approaches treating neurological changes associated with receptor trafficking.

## Methods

All animal experiments were conducted following the guidelines of the Canadian Council on Animal Care and the NIH, and approved by the Animal Care Committee at University of Toronto and Animal Care and Use Committee at Florida State University.

### Tissue culture, cDNA construction and transfection

Methods for primary neuron culture and HEK293 cell culture have been described previously [20, 83, 84]. Briefly, a block of cortical or hippocampal tissue was dissected from Wistar rat embryos (18 days gestation) at 4 °C. For biochemical experiments, a high density of dissociated cortical or hippocampal neurons (approximately 7 × 10^4^ cells/cm^2^) or HEK293 cells (6 × 10^4^ cells/cm^2^) was plated onto 60 mm culture dishes coated with poly-D-lysine. Neurons were cultured with Neurobasal™ media (Invitrogen, Grand Island, NY) supplemented with B27 (2 %), bFGF2 (2 ng/mL) and L-glutamine (0.5 mM), and HEK293 cells with Dulbecco’s Modified Eagles Media (DMEM) (Gibco-BRL, Grand Island, NY) supplemented with 10 % fetal bovine serum. For imaging experiments, a low density of cells (approximately 3 × 10^3^ cells/cm^2^) was cultured on glass cover-slips (MatTek, Ashland, MA). For electrophysiological recordings in neurons a low density culture of hippocampal neurons was plated in poly-D-lysine-coated dishes with Neurobasal™ media as mentioned above, or on collagen-coated dishes with minimum essential media (MEM) supplemented with 10 % horse serum. Since no difference was found in electrophysiological studies of neurons cultured with MEM versus those with Neurobasal™ media, the data were pooled. For electrophysiological recordings in HEK293 cells, low density culture was maintained in DMEM supplemented with 10 % fetal bovine serum.

Methods for constructing cDNAs of NMDAR subunits were described in our previous reports [31, 83]. The truncations in C-tail of the GluN2A or GluN2B subunit were carried out by introducing stops using site-directed mutagenesis. The mutation of serine to phenylalanine was performed to identify phosphorylation site(s) in the GluN2A subunit. For cDNA transfection HEK293 cells were incubated with LipoD293™ (SignaGen, Rockville, MD) and cDNAs of PSD95 (0.4 μg), GluN1-1a (1–2 μg) and GluN2A or GluN2A mutants (2–4 μg), or GluN2B or GluN2B mutants (2–4 μg). After a 5 h incubation with the cDNAs and transfection agents cells were incubated with DMEM supplemented with 10 % fetal bovine serum and AP5 (500 μM) and maintained for 48 h before experiments.

### Surface protein internalization assay

Surface protein internalization induced by bath treatment with NMDA (1 mM) and glycine (100 μM) (defined as high NMDA/glycine in this article) for 10 min or with DHPG (50 μM) for 5 min were determined with biochemical assays and imaging observations. For biochemical assays, cell surface proteins were biotinylated with cell impermeant non-cleavable EZ-Link Sulfo-NHS-LC-LC-Biotin (Pierce, Appleton, WI), as described previously [85, 86]. In brief, cultured cells without treatment or treated with vehicle (the standard extracellular solution for electrophysiological recordings, see below), high NMDA/glycine (for 10 min) or DHPG (for 5 min) at 37 °C were transferred onto ice and washed three times with ice-cold PBS and incubated with ice-cold PBS containing 1 mg/ml biotin for 1 h at 4 °C. The unbound biotin was removed through three washes with ice-cold PBS. Cells were then harvested in 1.5 ml of ice-cold PBS with a rubber policeman and lysed in 400 μl of ice-cold cell lysis buffer containing 50 mM Tris–HCl (pH 7.6), 150 mM NaCl, 1 % Igepal CA-630, 0.5 % sodium deoxycholate, 0.1 % SDS, 2 mM EDTA, 1 mM sodium orthovanadate, and the protease inhibitors Pepstatin A (20 μg/ml), Leupeptin (20 μg/ml), Aprotinin (20 μg/ml) and Phenylmethylsulfonyl fluoride (1 mM). The homogenates were centrifuged at 20,000× *g* for 5 min at 4 °C. Then, the supernatant (300 μl) was incubated with an NMDAR subunit antibody or with NeutrAvidin agarose (Thermo Scientific, Rockford, lL) to precipitate NMDAR subunit protein or biotinylated protein. NMDAR proteins expressed on cell surface were determined through detecting biotinylated NMDAR subunit protein in NMDAR immunoprecipitates or NMDAR proteins in pull downed proteins with NeutrAvidin agarose.

For image analysis, neurons cultured on glass cover-slips were biotinylated as mentioned above. Following treatment with the vehicle, high NMDA/glycine (for 10 min) or DHPG (for 5 min) at 37 °C, neurons were incubated with biotin for 1 h at 4 °C. And then neurons were fixed with ice-cold 4 % paraformaldehyde, permeabilized with 0.1 % Triton X-100 and incubated with an antibody against the GluN1 (1:500, BD Pharmingen), GluN2A (1:1000, Rabbit, Santa Cruz), or GluN2B (1:500, Rabbit, Invitrogen) subunit. Biotinylated neuronal surface proteins were visualized with streptavidin-conjugated Alexa 488 (green), and NMDAR subunit proteins were visualized with Alexa 568-conjugated goat anti-mouse or Donkey anti-rabbit IgG. Background staining was removed by thorough washing with PBS. Coverslips were then mounted using Fluoromount® (Sigma). Z-stack images of fluorescence staining were taken under an Axiovert 200 M fluorescence microscope (Carl Zeiss) equipped with CCD camera (Orca-ER, Hamamatsu) using software AxioVision 4.5 (Carl Zeiss). Co-localization of staining of the GluN1, GluN2A or GluN2B subunit with biotinylated cell surface proteins was analyzed using NIH-ImageJ software (NIH, Bethesda, ML) [87]. Ratios of pixel numbers showing the co-localized GluN1, GluN2A, or GluN2B subunit versus total GluN1, GluN2A or GluN2B subunit were calculated, respectively.

### Synaptic plasma membrane (LP1) preparation

To determine the effect of NMDAR endocytosis on phosphorylation of surface NMDAR proteins, the synaptic plasma membrane (LP1) [24, 30] was isolated as described previously [31]. In brief, cultured neurons were harvested and homogenized in ice cold homogenization buffer containing (mM): sucrose (320), HEPES (4, pH 7.4), NaF (50), sodium pyrophosphate (10, EMD), sodium glycerophosphate (20, Calbiochem), sodium orthovanadate (1), PMSF (0.1), aprotinin (20 μg/ml, MP), leupeptin (20 μg/ml, MP), and pepstatin (20 μg/ml, MP). The homogenate was spun at 1000x *g* for 15 min at 4 °C to remove the nuclear pellet and other large debris (P1). The supernatant (S1) was collected for further experiments to yield the crude synaptosomal fraction (P2) which was subsequently lysed by hypoosmotic shock in 0.5 ml of ice cold 4 mM HEPES (pH 7.4) plus protease/phosphatase inhibitors and three strokes of an eppendorf tube homogenizer. It was kept on ice for 30 min to ensure complete lysis. By using a pasteur pipette, the lysate was layered on top of a discontinuous gradient containing 0.8 to 1.0 to 1.2 M sucrose (top to bottom) in a clear tube, and was centrifuged at 150,000x *g* for 2 h at 4 °C. LP1 was recovered in the layer between 1.0 and 1.2 M sucrose, and then it was diluted to 0.32 M sucrose by adding 2.5 volume of 4 mM HEPES pH 7.4. The synaptic plasma membrane was pelleted by centrifugation at 150,000 g for 45 min at 4 °C and then resuspended in cell lysis buffer (mM): Tris–HCl (50, pH 7.4), NaCl (150), 1 % Igepal CA-630, 0.5 % deoxycholate, 0.1 % SDS, EDTA (2), NaF (50), sodium pyrophosphate (10), sodium glycerophosphate (20), sodium orthovandate (4); pepstatin A (20 μg/ml), leupeptin (20 μg/ml), aprotin (20 μg/ml), PMSF (1). The protein concentration was determined by Bradford assay. NMDAR subunit proteins were immunopurified from the LP1 fraction and their serine phosphorylation was examined by anti-pSer antibody (Invitrogen) in Western blot analysis.

### Immunoprecipitation and western blot

Immunoprecipitation and Western blotting were performed as described previously [31, 83]. Solubilized proteins (cell lysate or LP1 extracts) were incubated with antibodies overnight at 4 °C, followed by incubation with 25 μl of protein A-sepharose beads (Amersham Biosciences, Piscataway, NJ) for 3 h at 4 °C. The immunoprecipitates were washed 3 times with ice-cold cell lysis buffer, resuspended in 2X Laemmli sample buffer (120 mM Tris–HCl pH6.7, 4 % SDS, 10 % glycerol, 0.04 mg/ml bromophenol blue, 5 % 2-mercaptoethanol), and boiled for 5 min. Antibodies used for immunoprecipitation were: anti-GluN1 (mouse, 0.5 μg, BD Bioscience, San Jose, CA), anti-GluN2A C-terminal (rabbit, 1.5 μg, EMD Millipore, Billerica, MA), anti-GluN2A N-terminal (rabbit, 2 μg, Santa Cruz Biotech., Santa Cruz, CA), anti-GluN2B N-terminal (rabbit, 0.5 μg, Invitrogen) and anti-PKD1 (rabbit, 1 μg, Cell Signaling, Danvers, MA). To pull down biotinylated protein homogenates were centrifuged at 15,000x *g* for 15 min. 20 μl of supernatant was used to measure total GluN1, GluN2A or GluN2B. 260 μl of supernatant was incubated with 100 μl of 50 % NeutrAvidin agarose (Thermo Scientific) at 4 °C for 3 h. The agarose beads were washed three times with cell lysis buffer and the bound biotinylated proteins were eluted from the beads in 40 μl of 2 × Laemmli sample buffer by boiling.

The majority of the gels used for the Western blot analysis were 7.5 % polyacrylamide SDS-PAGE gels with Tris-glycine running buffer. Synthetic peptides of S1407-H1419 or S1407-H1419 (pS1416) were loaded on precast 4–12 % Bis–Tris polyacrylamide gels with MES running buffer (Invitrogen, Inc.) for the analysis of the specificity of the anti-pS1416 antibody. The filters were stripped repeatedly and probed successively with various antibodies. Antibody staining bands were visualized with Enhanced Chemiluminescence (Bio-Rad, Hercules, CA) or Immobilon (EMD Millipore). The band intensities were quantified with NIH-imageJ software (NIH, Bethesda, ML). Antibodies used in this study include anti-GluN1 (1:2000, mouse, BD Pharmingen), anti-GluN2A C-terminal (1:1000, rabbit, EMD Millipore), anti-GluN2A N-terminal (1:1000, rabbit, Santa Cruz), anti- GluN2B (1:1000, rabbit, Invitrogen), anti-pSer (1:1000, rabbit, Invitrogen), anti-pS1416 (1:1000, rabbit), anti-Kv3.1 (1:500, rabbit, BD Bioscience), anti-actin (1:4000, mouse), and antibodies recognizing the phosphorylated S916 in PKD1 [56] (1:1000, rabbit), phosphorylated T197 in PKA [55] (1:1000, rabbit) and phosphorylated T638 and T641 in PKC-α and β [51] (1:1000, rabbit), phosphorylated S606 in PKC-β [51] (1:1000, rabbit), phosphorylated T538 in PKC-θ [52] (1:1000, rabbit), phosphorylated T410 and T403 in PKC-ζ and λ [53, 54] (1:1000, rabbit) were used to examine the activation status of these enzymes, respectively. All these antibodies were purchased from the company, Cell Signaling.

To control variations which may occur from one experiment to another, samples treated only with culture medium were always tested as controls in each (or each repeat) biochemical experiment. We conducted careful, unbiased, densitometry analysis of all western blots by using NIH-ImageJ software. The relative densitometric intensities on the Western blots of the treated samples compared to samples treated only with culture medium were calculated, and multiplied by 100, as “Percent of Control”. These values were used to show the effects of any treatment. Ratios of the protein band intensities of the biotinylated GluN1, GluN2A or GluN2B subunit detected with streptavidin versus that with the antibodies were calculated to determine the amount of surface NMDARs and normalized to the ratio detected in cells treated only with culture medium conducted in parallel. The immunoprecipitation experiments from non-biotinylated tissues were conducted as negative controls.

### Lentivirus production and neuronal infection

Lentiviral particle production and infection were performed as recommended by the manufacture (Open Biosystems/Thermo Fisher Scientific, Pittsburgh, PA). For Lentiviral particle production, HEK293T cells were co-transfected with the Trans-Lentiviral packaging mix and the transfer vector containing green fluorescent protein (GFP) cDNA and the PKD1 shRNA (TGCTGTTGACAGTGAGCGCATCGTTCACTGTGACCTCAAATAGTGAAGCCACAGATGTATTTGAGGTCACAGTGAACGATATGCCTACTGCCTCGGA) or control shRNA (TGCTGTTGACAGTGAGCGATCTCGCTTGGGCGAGAGTAAGTAGTGAAGCCACAGATGTACTTACTCTCGCCCAAGCGAGAGTGCCTACTGCCTCGGA) (Open Biosystems/Thermo Fisher Scientific) as recommended by the manufacture (Open Biosystems/Thermo Fisher Scientific). The virus-containing supernatants were collected 48–72 h post-transfection, centrifuged and filtered through 0.45 μm low protein binding filter with the approximate titer 1 × 10^6^ transducing units per ml (Open Biosystems/Thermo Fisher Scientific). The viral stock was aliquoted and stored at −80 °C. Nine to ten days after plating, cultured neurons were infected by adding the viral-supplemented medium (stock) into culture medium (for biochemical experiments: 1.25 ml/ml; for electrophysiological recordings: 0.03 ml/ml). Four to five hours after infection, neurons were washed with 1X PBS and cultured for 7–10 days before electrophysiological or biochemical experiments. According to image observation more than 80 % of cells were infected.

### Electrophysiological recordings

Methods for recordings of NMDA-evoked single-channel or whole-cell currents, and mEPSCs have been described previously [20, 59, 83, 84]. For single-channel recordings, a standard extracellular solution was used for all recordings conducted in cultured neurons, containing (in mM): Na_2_SO_4_ (100), Cs_2_SO_4_ (10), CaCl_2_ (4.8), HEPES (25), glucose (32), TTX (0.001), glycine (0.003), bicuculline (0.01) and strychnine (0.01), pH: 7.35 and osmolarity: 310–320 mOsm. Free Ca^2+^ concentration in this solution was at 1.2 mM as confirmed by measurement with a Ca^2+^ selective electrode (Thermo Electron Corporation, Beverly, MA). To prevent cell damage during NMDAR activation, in this solution, Cs^+^ and SO_4_^2−^ replaced K^+^ and Cl^−^, respectively [88, 89]. Recording electrodes were pulled to a diameter of 1–2 μm at the tip and filled with the same extracellular solution but containing NMDA (0.01 or 1 mM as indicated) to evoke NMDAR-mediated single-channel currents (DC resistance: 8–10 MΩ). For electrophysiological recordings, the cultures were placed in a recording chamber on an inverted microscope (Axiovert S200 TV, Carl Zeiss, Oberkochen, Germany) equipped with 64 × Differential Interference Contrast (DIC) System. The image was magnified further by 30 x and displayed on a 17” TV monitor. The morphology of the cell soma and major processes of neurons were monitored. None of the bath solutions or experimental manipulations produced significant changes in size or shape of the cell soma and/or main processes.

Single-channel currents were recorded with a patch potential of 70 mV from the reversal potential using an AxoPatch 200B amplifier. Recorded electronic signals were filtered at 10 KHz (−3 dB, 8 pole Bessel) and sampled continuously onto a computer at 20 KHz with the pClamp9 software (Molecule Device, Sunnyvale, CA). Consistent with previous observations [59, 90], most (80 %) recorded channels from neurons bathed with the standard extracellular solution have main conductance levels ranging from 40–60 pS, and for the remainder, the main conductance levels were in the range of 10–30 pS. The recorded currents could be abolished by bath application of the lipophilic NMDAR channel blocker, MK-801 (2 μM), confirming that NMDAR-mediated currents were recorded. [20, 59]. Since only one main conductance level of channel openings was found in most patches, the channel open probability and duration of channel openings and closings were determined off-line by using a 50 % crossing threshold. The distribution histograms of the channel open and shut dwell-time (not including those ≤ 0.1 ms) were binned (log bin density = 9) and respectively fitted using Levenberg-Marquardt least-squares method in pSTAT6 software (Molecule Device). The usefulness of adding exponential components was assessed using an F-test and also by visual inspection. We calculated the durations of bursts, clusters and super-clusters of channel openings [91], which were identified according to critical times (t_c_) where each t_c_ was defined by 1-e^-tc/τs^ = e^-tc/τm^ (τs and τm: the time constants of short and intermediate gap lengths) [59, 90].

To examine effects of DHPG application, after a 3 to 5 min period of control recordings, DHPG (50 μM) was bath applied for 5 min and then recordings were continued for another 10 to 20 min. To examine effects of high NMDA/glycine application, the extracellular solution containing 1 mM NMDA was applied after the cell-attached configuration was formed. Control recordings were conducted for 3–5 min in neurons bathed with the extracellular solution containing 1 mM NMDA, and then glycine (100 μM) was bath co-applied.

For whole-cell voltage-clamp recordings cells were bathed with the same standard extracellular solution mentioned above except that 10 μM glycine was applied for recordings in HEK cells. Recording electrodes were filled with intracellular solution composed of (in mM): CsCl (145), BAPTA (0.5), HEPES (10), MgCl_2_ (2), K-ATP (4), osmolarity 290–300 mOsm (DC resistance: 4–7 MΩ). NMDA-mediated whole-cell currents were evoked by application of 1 mM NMDA dissolved in the extracellular solution via a computer-controlled multi-barrel fast-step perfusion system (SF-77B perfusion fast-step system, Warner Instrument, Hamden, CT). Whole-cell currents were recorded using Axopatch 200B amplifiers (Molecular Devices, Sunnyvale, CA) under the voltage-clamp condition at a holding potential of −60 mV except where indicated. On-line data acquisition and off-line analysis were performed using pClamp9 software (Molecular Devices). To determine effects produced by intracellular delivery of PKD1 added in the intracellular solution filling recording electrodes, recorded whole-cell currents evoked by NMDA application were compared with that recorded immediately after breakthrough (defined as “0” min in this work).

Miniature excitatory post-synaptic currents (mEPSCs) were recorded in cultured hippocampal neurons (following the protocol shown in Fig. 7e; Additional file 8: Figure S8A, D and G) under the voltage-clamp condition at a holding potential of −60 mV. On-line data acquisition and off-line analysis were performed using pClamp9 software (Molecular Devices). The threshold for detection of mEPSCs was approximately −4 pA, which was determined with an all-point histogram assay, and optimized for each cell so as to collect > 95 % of mEPSCs [83]. NMDAR-mediated mEPSCs were identified by application of the NMDAR antagonist AP5 (100 μM). Non NMDAR-mediated mEPSC frequency and amplitude were analysed with software Mini Analysis Program (Synaptosoft, Decatur, GA). To determine charges (Q) mediated by NMDARs and non-NMDARs, mEPSCs were averaged after alignment of the rising edge using WinWCP software (Strathclyde Institute of Pharmacy and Biomedical Sciences, Glasgow, UK). Traces with noise artifacts or with more than one mEPSC per 200 ms recording period were not included. mEPSCs were averaged for each treatment period (at least 30 traces). The charges mediated by non-NMDARs and NMDARs were respectively calculated by integrating currents mediated by these receptors, and normalized to that measured before DHPG application.

In order to record NMDAR activity before, during and/or after regulated NMDAR endocytosis, which was induced by bath application of DHPG or high NMDA/glycine, only one neuron or one patch from one neuron in one culture dish was allowed to be tested. To sample an “n” of 5 or more cells for one group of experiments, at least 2 separated cultures were required. All chemicals used in this work were obtained from Sigma (St. Louis, MO) except for those as indicated.

### Statistic analysis

Multiple tests for examining normality or variance of data were performed to determine which type of statistic tests could be used to examine differences between experimental groups. For comparisons of channel activity before and/or after bath application of any agents, paired two-tailed Student’s t-tests were performed. For comparisons of protein expression and phosphorylation with that in the group of cells treated only with culture medium, independent two-tailed Student’s t-tests were performed. Repeated measures analysis of variance (ANOVA) were used for comparison of experiments involving time courses. All data are expressed as mean ± SEM.

## Additional files

Additional file 1: Figure S1. The endocytosis of NMDARs (the GluN1 subunit). (PDF 62 kb) Additional file 2: Figure S2. The endocytosis of NMDARs (the GluN2A and GluN2B subunits). (PDF 280 kb) Additional file 3: Figure S3. The endocytosis of GluN1-1a/GluN2B, but not GluN1-1a/GluN2A, receptors was prevented by C-tail truncation after aa 857. (PDF 90 kb) Additional file 4: Figure S4. NMDAR internalization causes an increase in phosphorylation of the GluN2A subunit at S1416. (PDF 100 kb) Additional file 5: Figure S5. An antibody selectively recognizes phosphorylation of the GluN2A subunit at S1416. (PDF 51 kb) Additional file 6: Figure S6. PKD1 phosphorylates and down-regulates NMDARs. (PDF 119 kb) Additional file 7: Figure S7. Infection of PKD1 shRNA reduces PKD1 expression. (PDF 103 kb) Additional file 8: Figure S8. Effects of PKD1 knockdown on glutamate-mediated mEPSCs in hippocampal neurons. (PDF 78 kb)

## Abbreviations

aa: amino acid
AMPAR: α-amino-3-hydroxy-5-methyl-4-isoxazolepropionic acid receptor
ANOVA: repeated measures analysis of variance
AP5: (2*R*)-amino-5-phosphonovaleric acid; (2*R*)-amino-5-phosphonopentanoate
BAPTA: 1, 2-bis(o-aminophenoxy)ethane-N,N,N’,N’-tetraacetic acid
bFGF2: BFibroblast growth factor-basic recombinant protein
*B*_*s*_: the duration of bursts
DHPG: (*S*)-3,5-Dihydroxyphenylglycine
EDTA: ethylenediaminetetraacetic acid
GluN1: glycine-binding NMDA receptor subunit 1
GluN2A: glutamate-binding NMDA receptor subunit 1
GluN2B: glutamate-binding NMDA receptor subunit 2
HEPES: 4-(2-hydroxyethyl)-1-piperazineethanesulfonic acid
HEK: human embryonic kidney
K-ATP: potassium-adenosine triphosphate
Kv: voltage-gated potassium channel
LTD: long-term depression
LP1: the synaptic plasma membrane
mEPSCs: miniature excitatory post synaptic currents
NMDA: *N*-Methyl-D-aspartic acid or *N*-Methyl-D-aspartate
PBS: phosphate-buffered saline
PKA: protein kinase A
PKC: protein kinase C
PKD: protein kinase D
PMSF: phenylmethylsulfonyl fluoride
*P*_*o*_: channel open probability
PSD95: postsynaptic density-95
pS1416: phosphorylated serine residue 1416
shRNA: small hairpin RNA
X-link: protein complex comprised of an antibody against the extracellular loop of the GluN1 subunit and a secondary antibody against mouse IgG
*t*_*o*_: mean open time
