# Supplementary figures and images for: Regulated internalization of NMDA receptors drives PKD1-mediated suppression of the activity of residual cell-surface NMDA receptors
Source: Mol Brain. 2015 Nov 19;8:75. doi: 10.1186/s13041-015-0167-1 (PMC4653853; doi:10.1186/s13041-015-0167-1)

**A**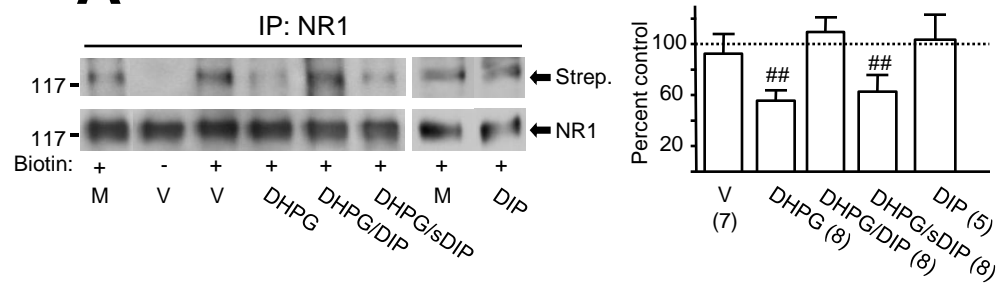**B**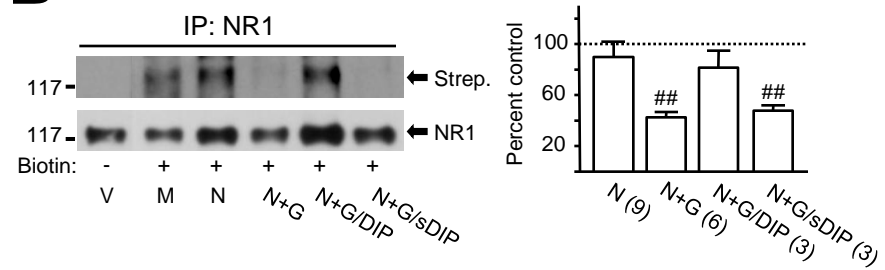

Supplement: Additional file 1: Figure S1. — The endocytosis of NMDARs (the GluN1 subunit). (PDF 62 kb) [file 13041_2015_167_MOESM1_ESM.pdf]

**A**

M

V

N+G

DHPG

Biotin

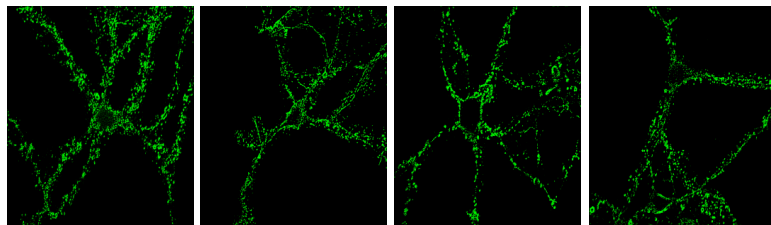

NR2A

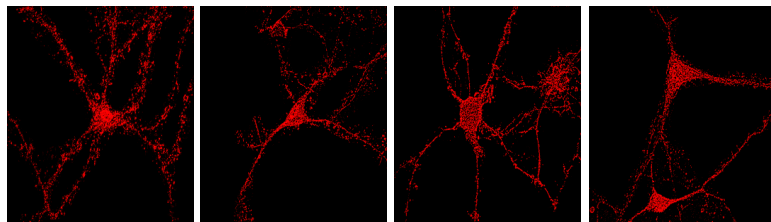

Co-local.

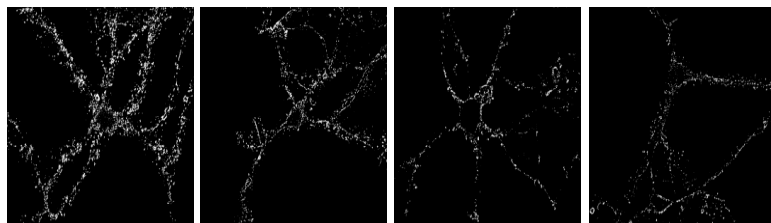

Merge

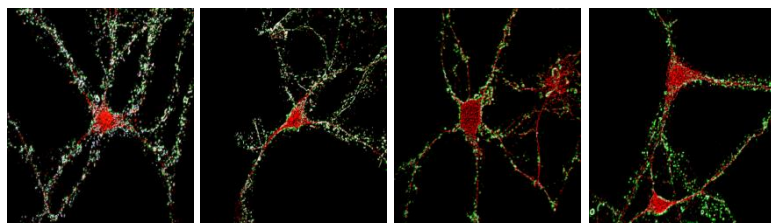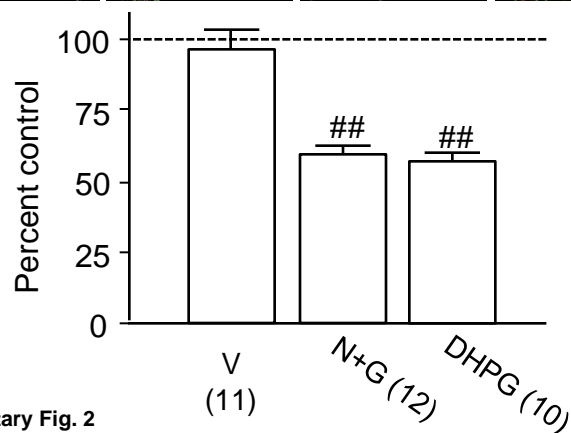**B**

M

V

N+G

DHPG

Biotin

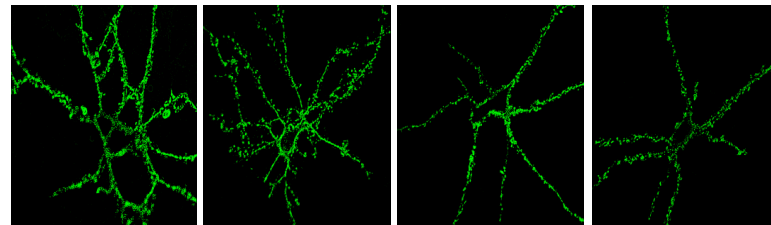

NR2B

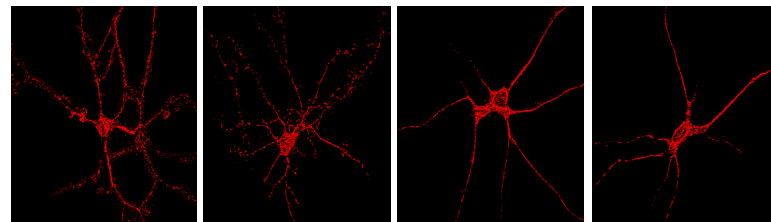

Co-local.

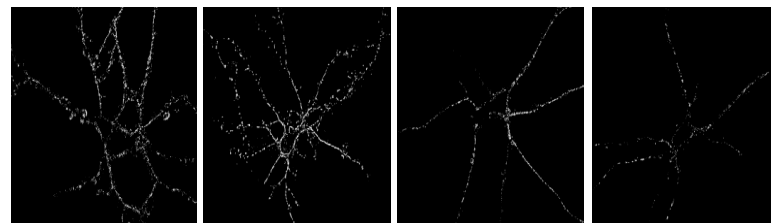

Merge

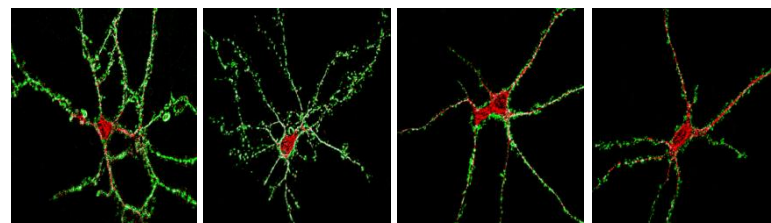40  $\mu$ m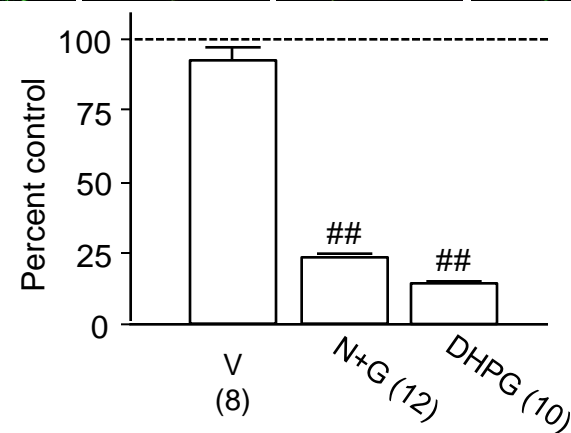

Supplement: Additional file 2: Figure S2. — The endocytosis of NMDARs (the GluN2A and GluN2B subunits). (PDF 280 kb) [file 13041_2015_167_MOESM2_ESM.pdf]

**A**

NR1-1a/NR2A

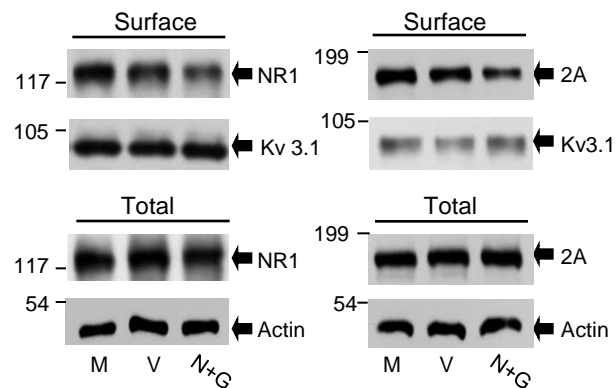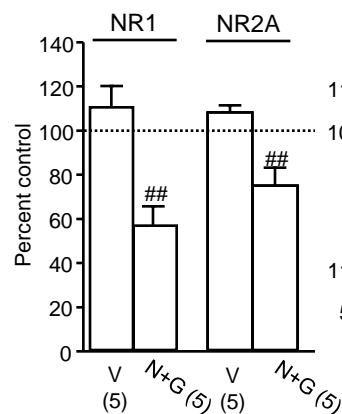**B**

NR1-1a/NR2B

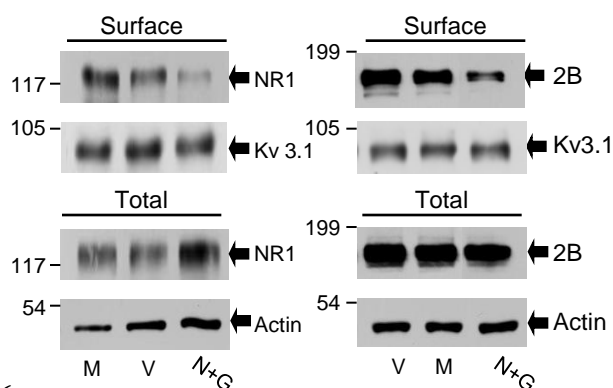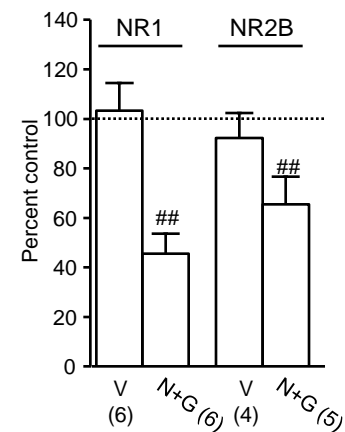**C**

NR1-1a/NR2A1-857

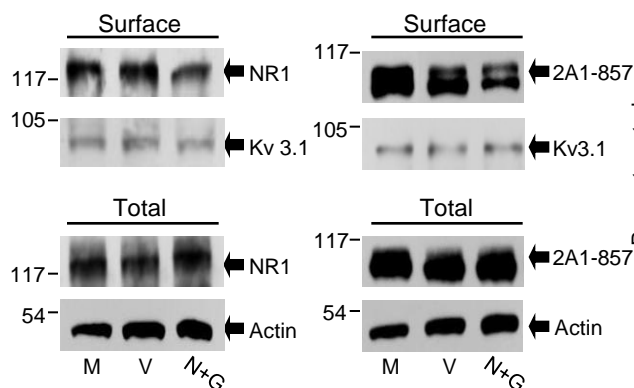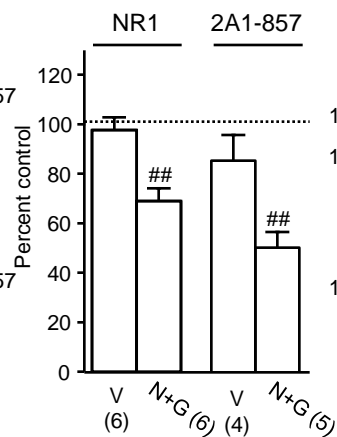**D**

NR1-1a/NR2B1-857

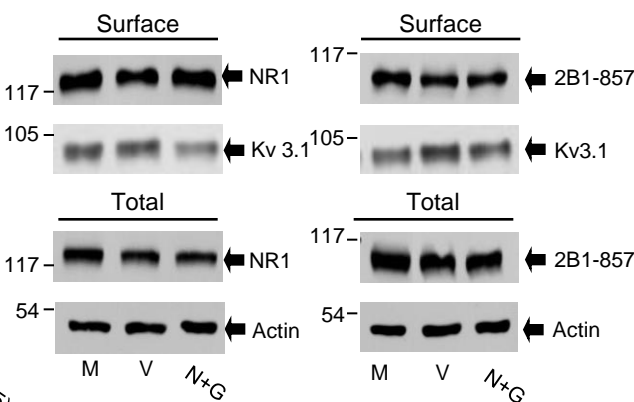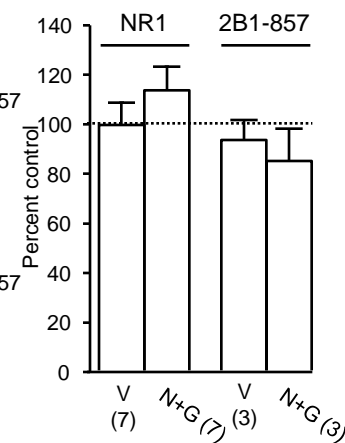

Supplement: Additional file 3: Figure S3. — The endocytosis of GluN1-1a/GluN2B, but not GluN1-1a/GluN2A, receptors was prevented by C-tail truncation after aa 857. (PDF 90 kb) [file 13041_2015_167_MOESM3_ESM.pdf]

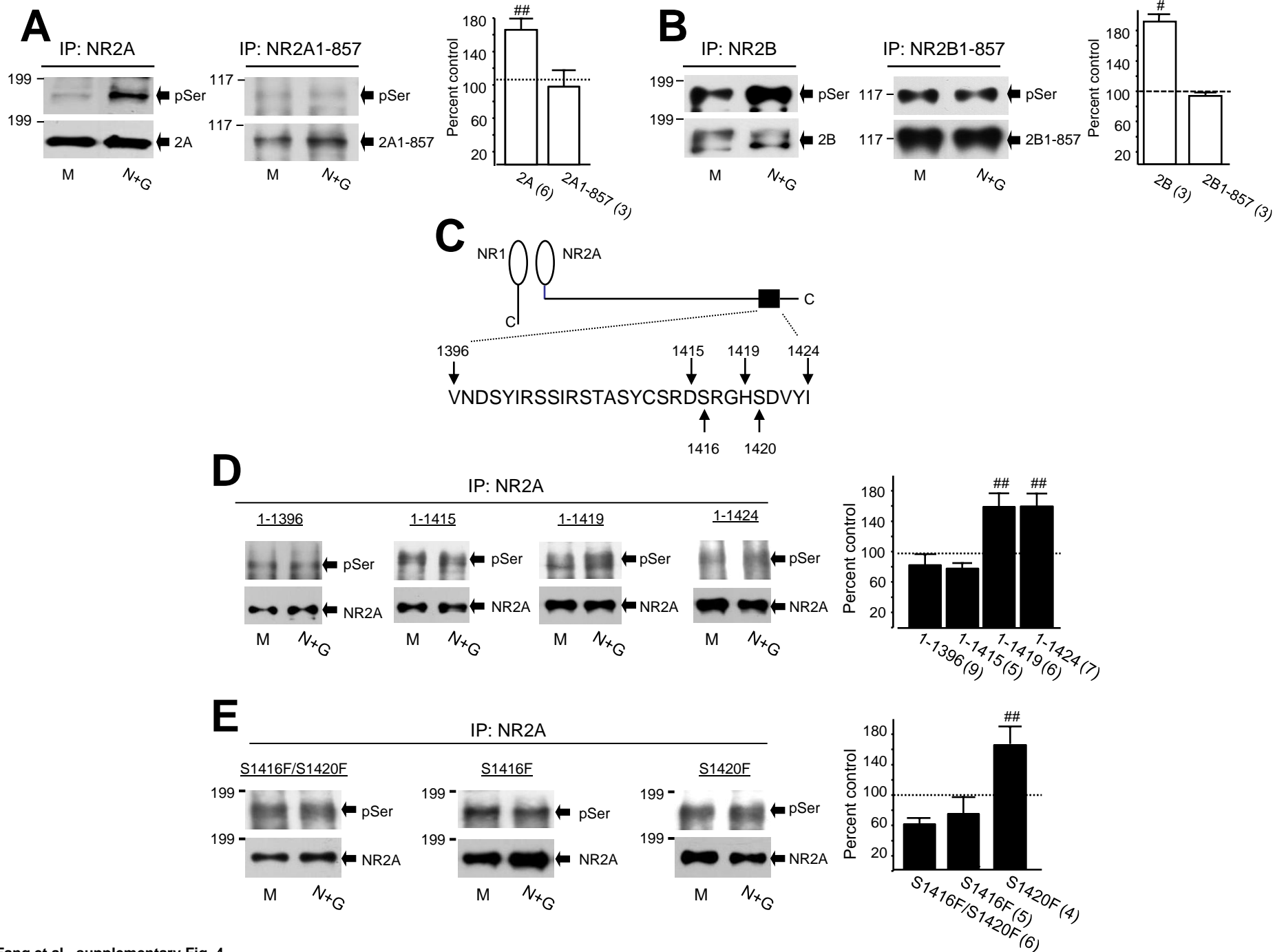

Supplement: Additional file 4: Figure S4. — NMDAR internalization causes an increase in phosphorylation of the GluN2A subunit at S1416. (PDF 100 kb) [file 13041_2015_167_MOESM4_ESM.pdf]

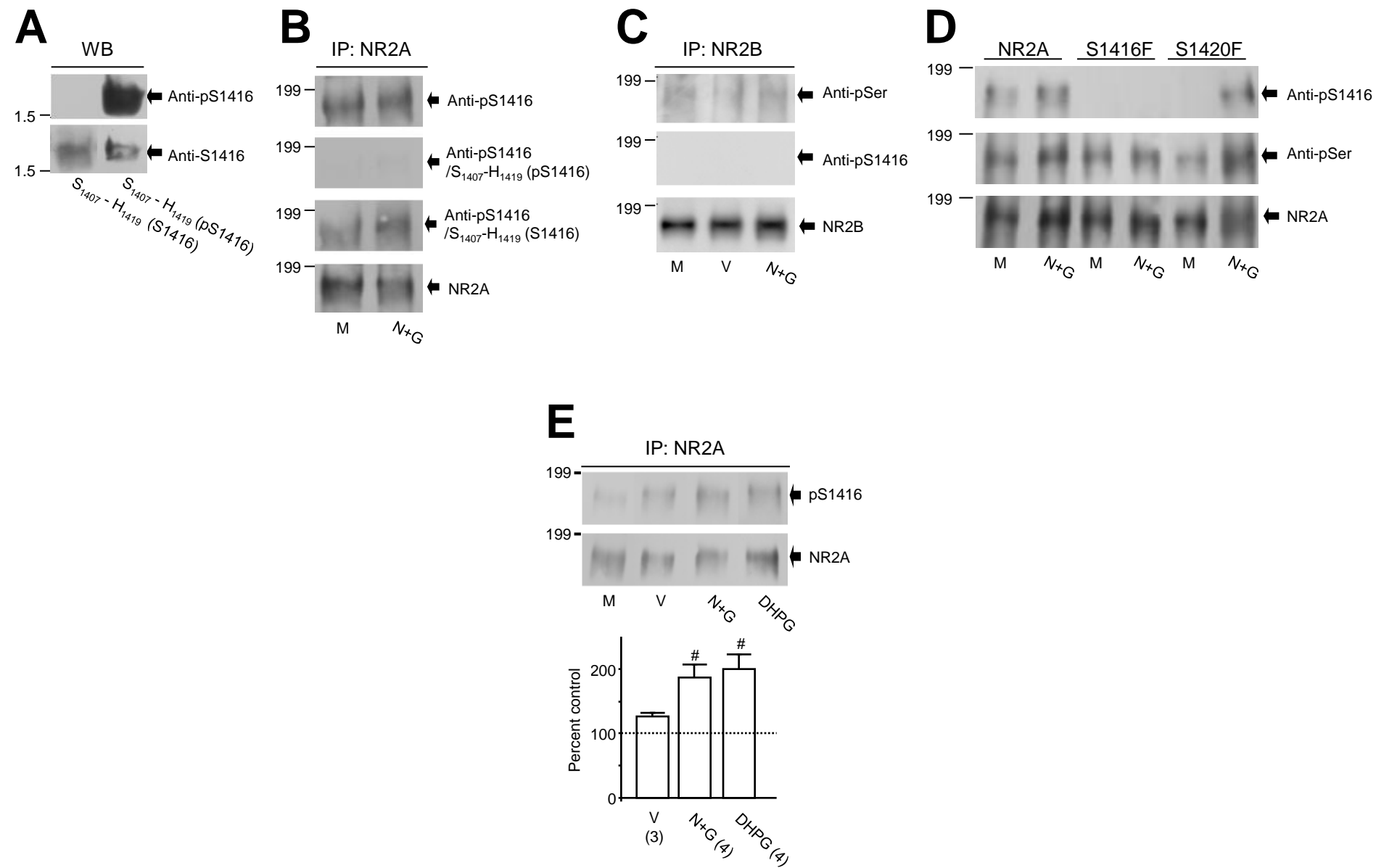

Supplement: Additional file 5: Figure S5. — An antibody selectively recognizes phosphorylation of the GluN2A subunit at S1416. (PDF 51 kb) [file 13041_2015_167_MOESM5_ESM.pdf]

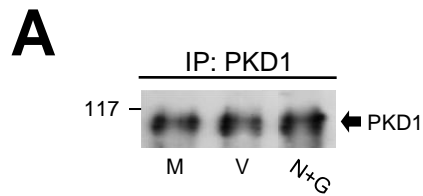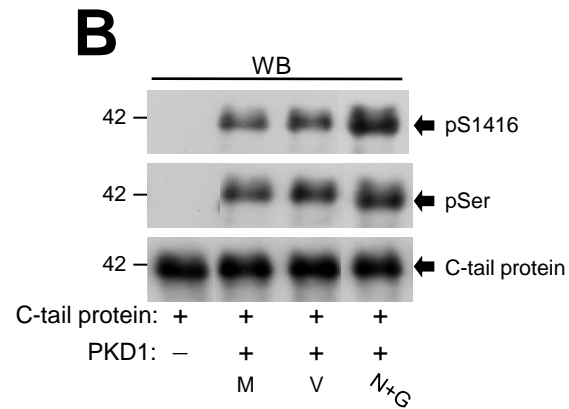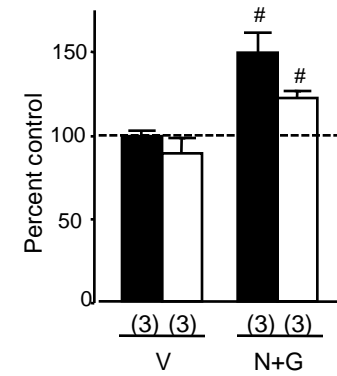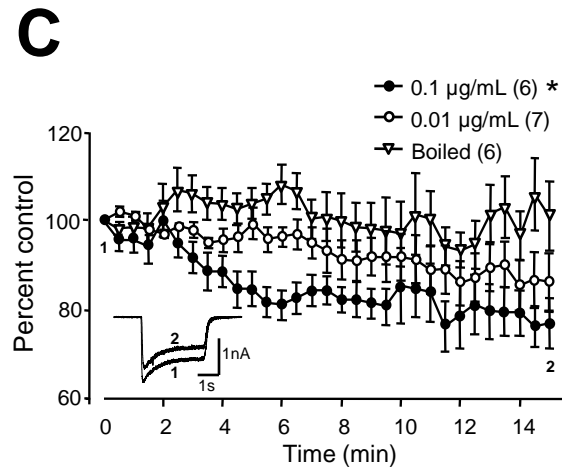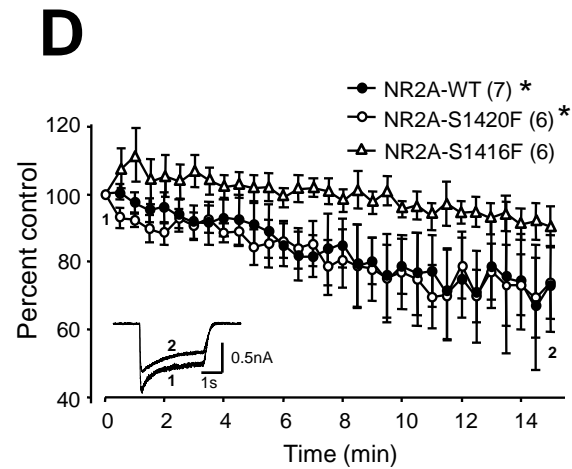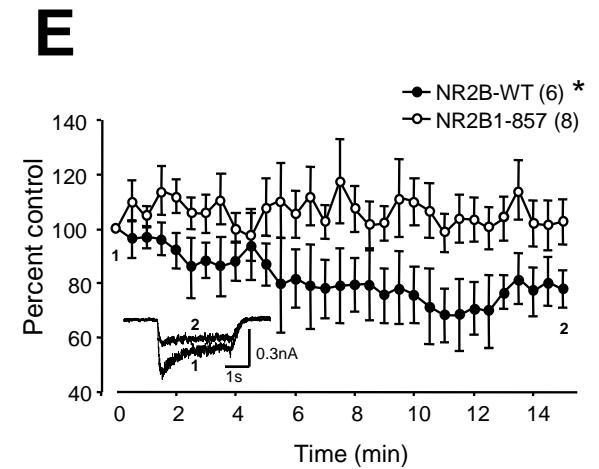

Supplement: Additional file 6: Figure S6. — PKD1 phosphorylates and down-regulates NMDARs. (PDF 119 kb) [file 13041_2015_167_MOESM6_ESM.pdf]

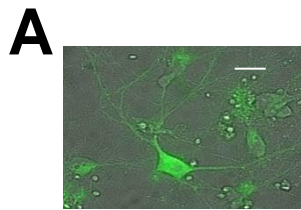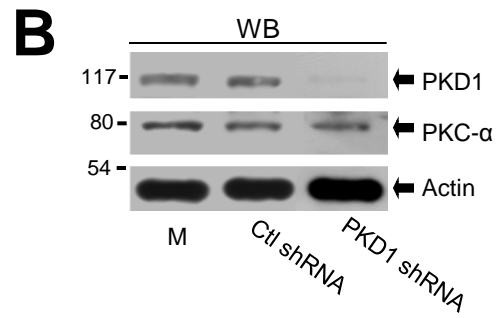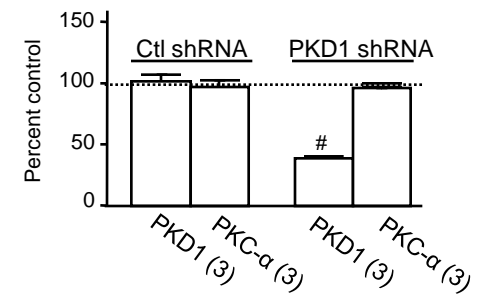

Supplement: Additional file 7: Figure S7. — Infection of PKD1 shRNA reduces PKD1 expression. (PDF 103 kb) [file 13041_2015_167_MOESM7_ESM.pdf]

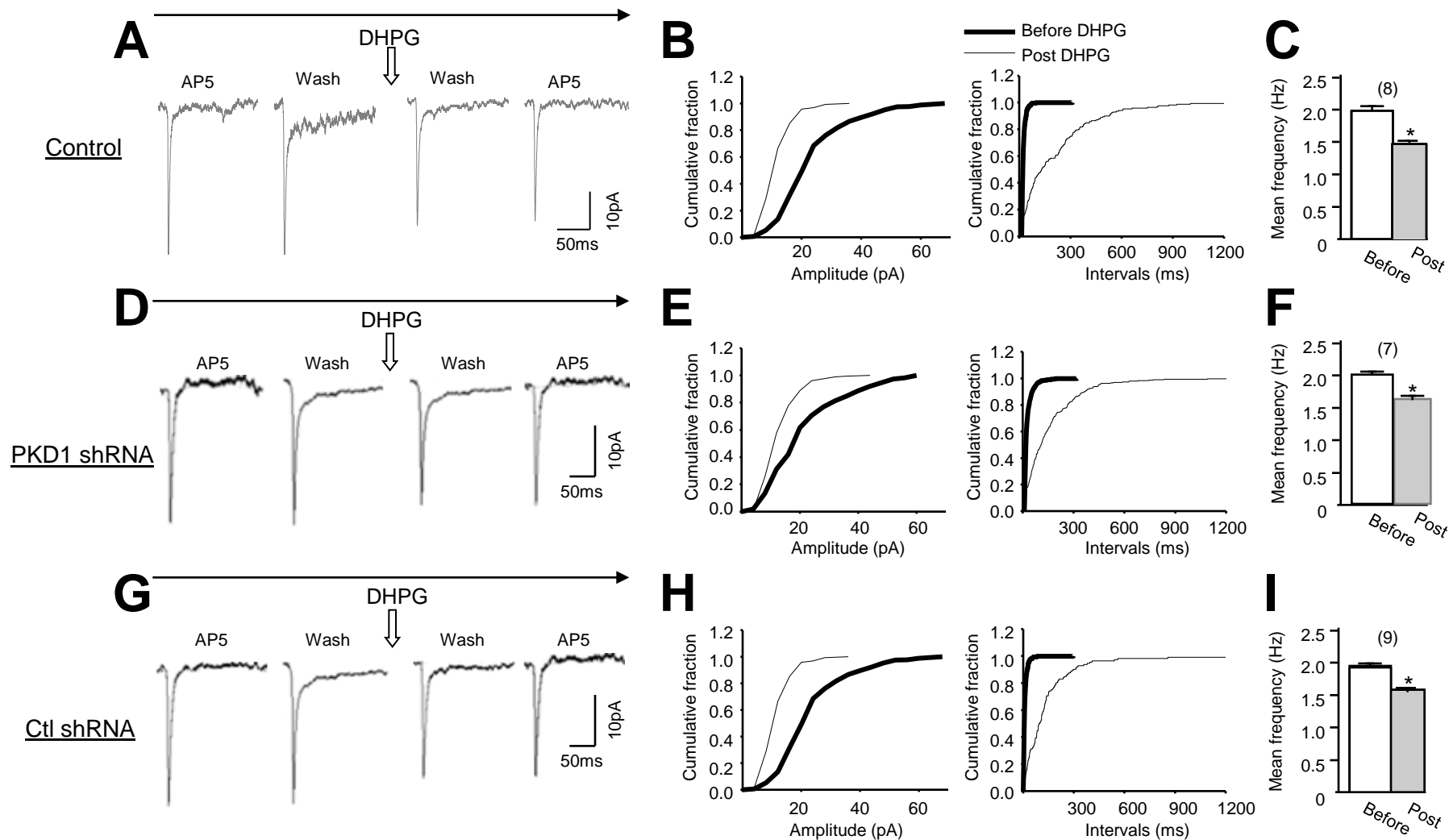

Supplement: Additional file 8: Figure S8. — Effects of PKD1 knockdown on glutamate-mediated mEPSCs in hippocampal neurons. (PDF 78 kb) [file 13041_2015_167_MOESM8_ESM.pdf]
